# Tofacitinib-Induced Antineutrophil Cytoplasmic Antibodies (ANCA)-Associated Vasculitis With Crescentic Glomerulonephritis

**DOI:** 10.7759/cureus.18663

**Published:** 2021-10-11

**Authors:** Uyioghosa Asemota, Sheldon Greenberg, Amit Gulati, Kamlesh Kumar, Kalyana Janga

**Affiliations:** 1 Internal Medicine, Maimonides Medical Center, Brooklyn, USA; 2 Nephrology, Maimonides Medical Center, Brooklyn, USA

**Keywords:** biopsy, antinuclear cytoplasmic antibodies, glomerulonephritis, crescentic, tofacitinib

## Abstract

Antineutrophil cytoplasmic antibodies (ANCA) vasculitis is common after the age of 50 years but it can occur at any age. There is a slight male preponderance and it is more common in Whites than Blacks but the black race confers a worse prognosis. The clinical features of ANCA vasculitis vary considerably. The manifestation of the disease depends on the organs affected, the chronicity of the disease, and how quiescent it is. Non-specific symptoms of malaise, fatigue, fever, and weight loss are common. Crescentic glomerulonephritis with focal necrosis is usually the pathology underlying renal disease. Manifestations of renal disease include hematuria and proteinuria which may progress to renal failure.

We present a case of a 75-year-old female who presented with acute worsening of renal function and nephrotic-range proteinuria with positive testing for p-ANCA after the recent commencement of treatment with tofacitinib. This prompted a suspicion of ANCA-vasculitis. The patient was started on pulse dose steroids and rituximab after kidney biopsy confirmation of ANCA-vasculitis with crescentic glomerulonephritis.

## Introduction

Tofacitinib is a specific inhibitor of Janus-associated kinases (especially JAK1 and JAK3). In the United States, it is approved for the treatment of moderate to severe active rheumatoid arthritis, psoriatic arthritis, and moderate to severe ulcerative colitis [1-3]. Janus kinases play important roles in immune activation, hematopoiesis, and cancer cachexia [3,4]. Multiple trials have shown the efficacy of tofacitinib either as monotherapy or in combination with disease-modifying anti-rheumatic drugs (DMARDs) [5-7]. However, the long-term utility of tofacitinib in the treatment of rheumatoid arthritis (RA) has been modest. One study in Canada showed that the median drug survival was two years and about 30% patients discontinued the drug due to lack of efficacy and another 27% discontinued it because of adverse effects [8]. Tofacitinib is dosed 5 mg twice daily or as 11 mg in extended-release form. Tofacitinib is metabolized in the liver through the Cytochrome 3A4 (CYP) pathway, it is, therefore, subject to drug-drug interactions with drugs that induce or inhibit CYP 3A4. Common adverse effects of tofacitinib include neutropenia, infections, diarrhea, and fatigue. The commonest side effect of tofacitinib therapy is infections and infestations and this is related to neutropenia [1]. The mechanism of neutropenia induction by tofacitinib is through the oxidation of tofacitinib to nitrenium ion by myeloperoxidase (MPO) in neutrophils, which reacts with sulfhydryl groups of cysteine residues of cellular proteins in leucocytes [9].

## Case presentation

We present a case report of a 75-year-old female who had started tofacitinib for the treatment of refractory rheumatoid arthritis. The patient had been unsuccessfully treated with adalimumab and etanercept with recurrence of symptoms after about a month of treatment with each of these medications. She had also been on variable doses of prednisone throughout the course of her treatment.

Two months after starting treatment with tofacitinib, her creatinine was noted to have increased from 1.9 mg/dL to 2.9 mg/dL together with 9 gm/day of proteinuria. At this time she also was p-ANCA positive though prior testing for p-ANCA was negative, four years ago. She was then referred to a nephrologist by her rheumatologist.

Tofacitinib was stopped and she was admitted to the hospital for acute kidney injury. She was started on pulse dose steroids, followed by 60 mg of prednisone daily. A kidney biopsy was done on admission which showed p-ANCA mediated pauci-immune focal necrotizing and focal sclerosing glomerulonephritis with 23% partial crescents. Rituximab was subsequently added to her treatment regimen and further serology workup was negative for anti-Smith antigen, anti-histone antigen, and anti-ribonucleoprotein antigen. ANA was positive and C3 was low.

She was readmitted a month after her first admission for shortness of breath, dyspnea on exertion, progressive leg swelling and treated for congestive heart failure and cardiorenal syndrome. A month after her second admission, she was readmitted for respiratory distress. On physical examination, she had tachycardia and tachypnea and had bilateral crackles at her lung bases with bilateral pitting pedal edema. Computerized tomography scan of the chest showed extensive bilateral interstitial pneumonitis which had progressed from a previous study. She was placed on pulse dose steroids and high flow oxygen and subsequently 40 mg of prednisone 12 hourly and IV Cyclophosphamide for interstitial pneumonitis secondary to ANCA-vasculitis. The treatment with Cyclophosphamide was complicated by the development of painful mouth ulcers. The patient was also placed on trimethoprim-sulfamethoxazole for Pneumocystis jirovecii pneumonia prophylaxis but switched to Atovaquone because of hyperkalemia. Although she was on high flow oxygen, the patient began to desaturate and the treatment course was complicated by bacterial infections and sepsis. Her oxygen saturation continued to get worse until she developed cardiopulmonary arrest and died.

## Discussion

Our patient presented with acute worsening of renal function and nephrotic-range proteinuria with positive testing for p-ANCA after the recent commencement of treatment with tofacitinib. This prompted a suspicion of ANCA-vasculitis. The patient was placed on pulse dose steroids and rituximab after kidney biopsy confirmation of ANCA-vasculitis with crescentic glomerulonephritis.

**Figure 1:**
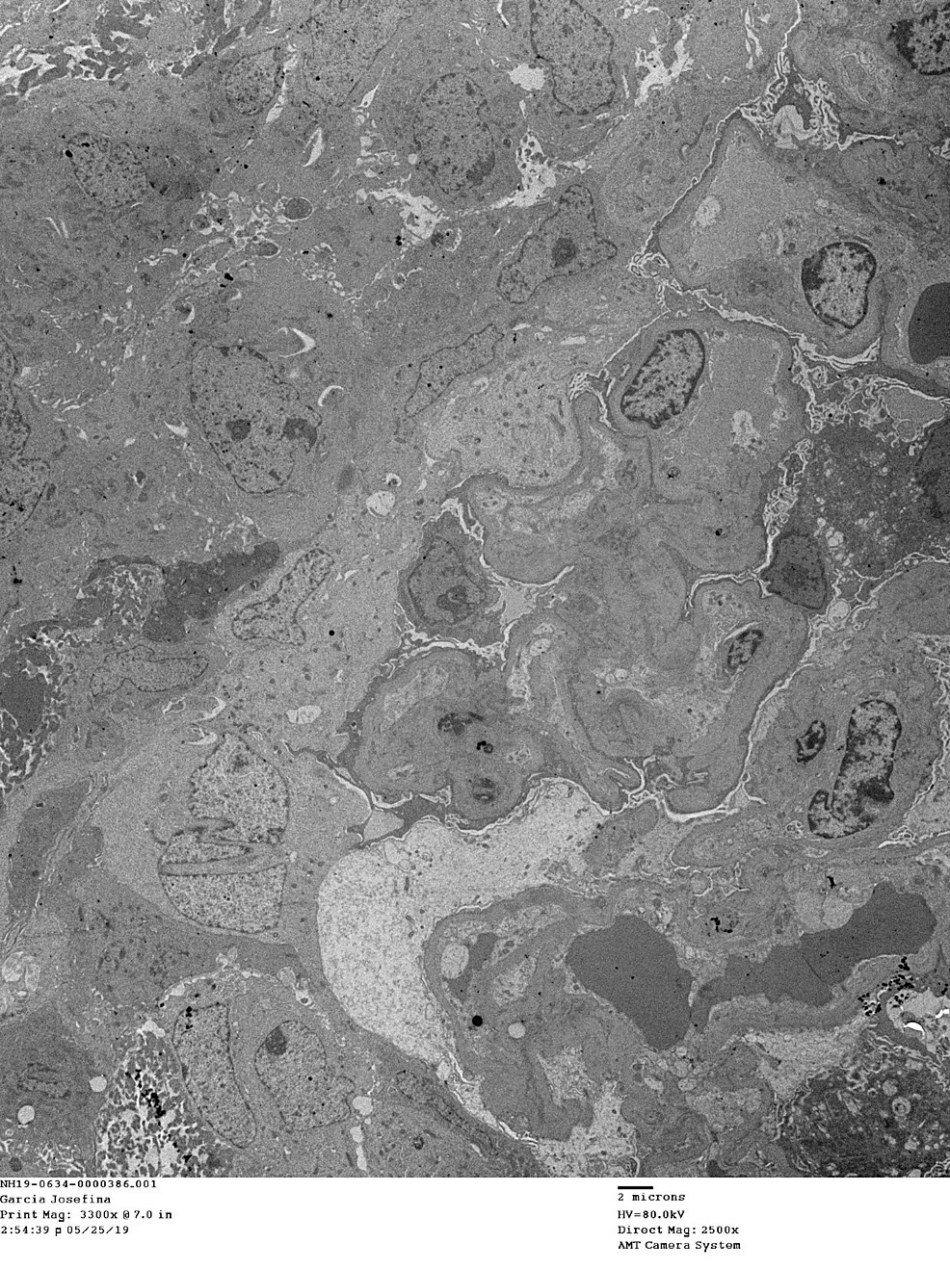
Electron microscopic image showing crescent formation

**Figure 2:**
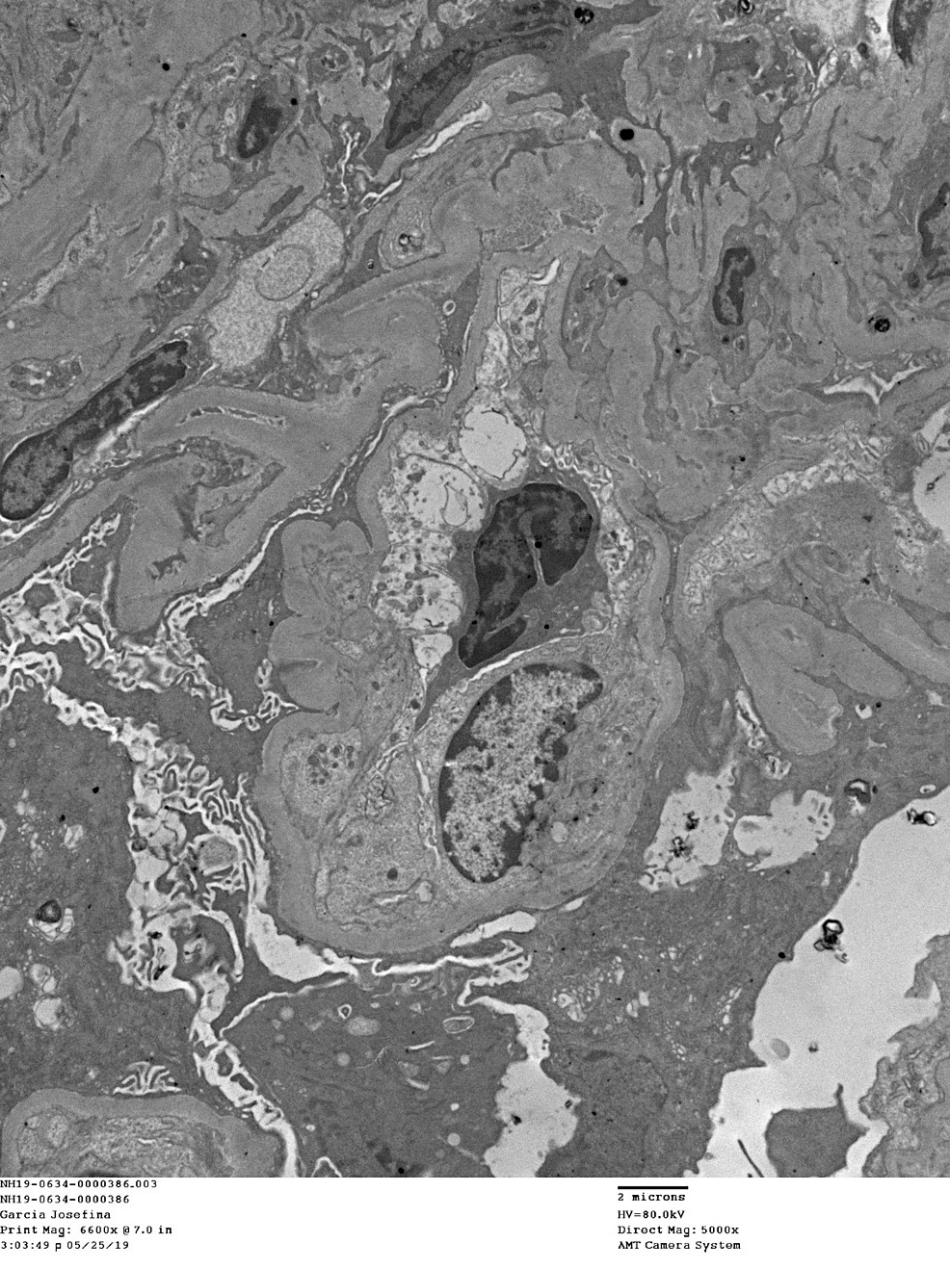
Electron microscopic image showing tuft winking

**Figure 3:**
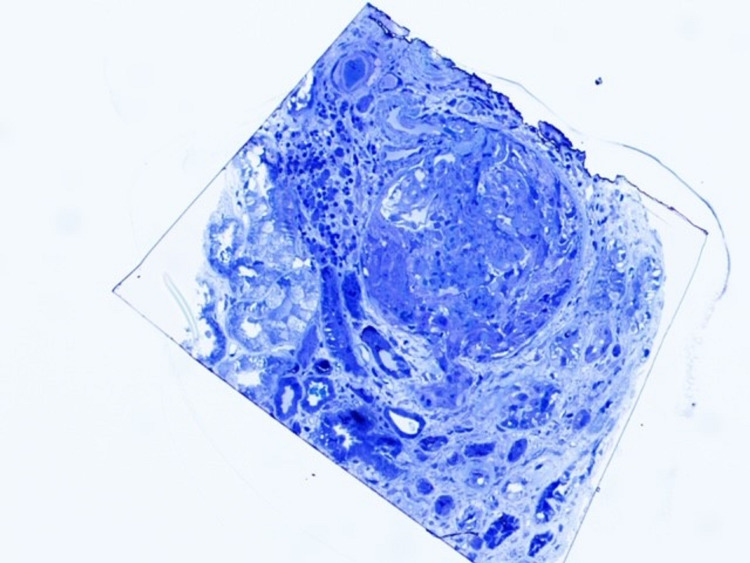
Toluidine blue stain showing crescent formation

**Figure 4:**
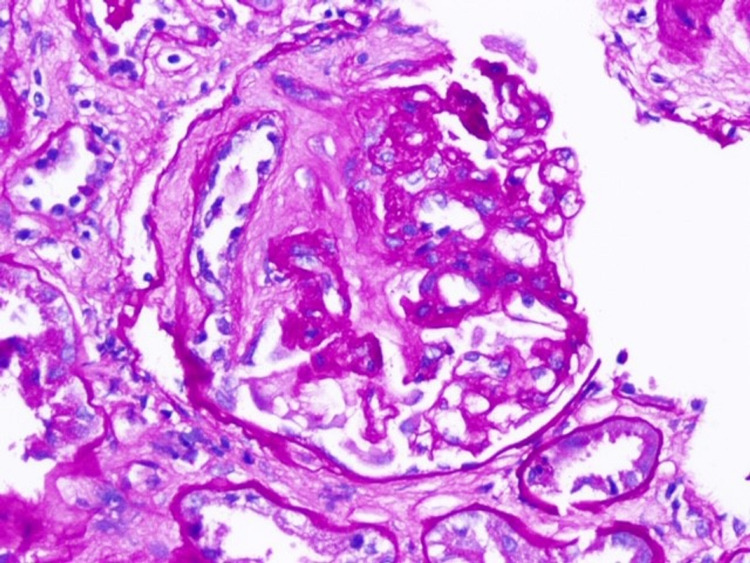
Periodic Acid Schiff (PAS) stain showing segmental sclerosis

**Figure 5:**
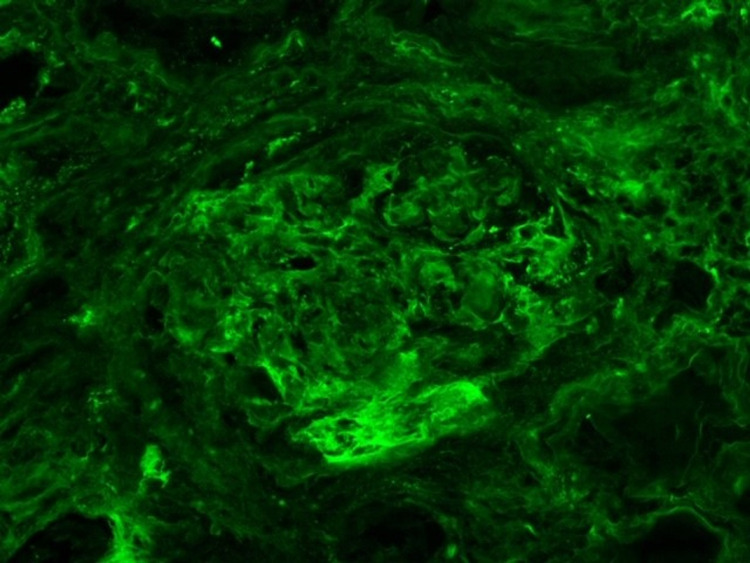
Fibrinogen immunofluorescence showing necrosis

ANCA-associated vasculitis (AAV) is one of the causes of small vessel vasculitis. Other causes of small vessel vasculitis include anti-glomerular basement membrane disease, IgA vasculitis, and cryoglobulinemic vasculitis. ANCA-associated vasculitis is pauci-immune necrotizing vasculitis with few or no immune deposits in the walls of small vessels affected. A distinguishing feature of AAV is the presence of necrotizing inflammation (Figures 1-5) [10-12].

AAV is categorized based on clinical and pathological characteristics into granulomatosis with polyangiitis (GPA), formerly known as Wegener’s granulomatosis, eosinophilic granulomatosis with polyangiitis (EGPA), and microscopic polyangiitis (MPA). Granulomatous inflammation is common to both GPA and EGPA, however, EGPA is associated with asthma and eosinophilia unlike GPA. Granulomas are absent in MPA. According to the revised International Chapel Hill Consensus Conference Nomenclature of Vasculitides, the classification of AAV should include ANCA-specificity, for example, PR3-ANCA or MPO-ANCA because this confers more specificity to the disease entity [13-15].

It is clear that ANCA plays a role in the pathogenesis of pauci-immune small vessel vasculitis as there is a strong correlation between the presence of antineutrophil cytoplasmic antibodies and disease development [15]. The development of ANCA in the blood is multifactorial as genetics, environmental factors and an inciting antigen are all contributory [12,15,16]. Most cases are idiopathic but the pathogenic potential of ANCA is strengthened by the onset of pauci-immune vasculitis with the appearance of ANCA in the sera of patients on certain culprit drugs. Propylthiouracil, hydralazine, and cocaine laced with levamisole are well-documented drug inducers of ANCA-associated vasculitis [17].

The relationship between ANCA titers and disease activity is unclear and not well understood. Though high ANCA titers usually correlate with worse disease activity, the absence of ANCA does not mean the absence of disease activity. However, ANCA-negative vasculitis is more often associated with renal-limited disease while ANCA serotype positivity is a strong marker for the underlying genetic pathogenesis [16]. It is also possible for individuals without any evidence of systemic vasculitis to test positive for ANCA, though this may be a harbinger for developing vasculitis in the future.

Although the relationship between ANCA titers and disease activity is unclear, the relationship between ANCA type and disease relapse is well established. PR3 ANCA confers double the risk of disease relapse compared to MPO-ANCA. Bone destruction or saddle-nose deformity is invariably associated with PR3-ANCA while most patients with kidney-limited disease are positive for MPO-ANCA. More than half of the patients with Churg-Strauss syndrome test negative for ANCA [14,16].

ANCA vasculitis is more common after the age of 50 years but it can occur at any age. There is a slight male preponderance and it is more common in Whites than Blacks but the black race confers a worse prognosis. The clinical features of ANCA vasculitis vary considerably. The manifestation of the disease depends on the organs affected, the chronicity of the disease, and how quiescent it is. Non-specific symptoms of malaise, fatigue, fever, and weight loss are common. Crescentic glomerulonephritis with focal necrosis is usually the pathology underlying renal disease. Manifestations of renal disease include hematuria and proteinuria which may progress to renal failure. Renal disease may also manifest as rapidly proliferative glomerulonephritis. Skin manifestations of vasculitis typically present as palpable purpura that begin as recurrent crops in the lower extremities which may become ulcerated. Necrotizing inflammation in the upper respiratory tract could lead to bone destruction and manifest as saddle-nose deformity. This is more common in patients who are PR3-ANCA positive [14]. Lung involvement is common, patients could present with hemoptysis from hemorrhage of the alveolar capillaries. Cardiac disease in AAV is not uncommon, presentation can range from heart block, infarction and pericarditis.

The diagnosis of AAV is based on clinicopathologic features. ANCA testing aids in the diagnosis but has some limitations. Testing for ANCA includes indirect immunofluorescence (IIF) assay and enzyme immunoassay (EIA). IIF shows two distinct patterns of staining. A diffuse pattern of staining throughout the cytoplasm and around the nucleus is described as cytoplasmic, c-ANCA and perinuclear, p-ANCA. Conventional practice has been to do initial testing by IIF and confirmation of positive ANCA by EIA, however recent studies have shown significant variability in results by standard tests for ANCA by IIA and there is now a trend to do ANCA screening by EIA methods only, especially because PR3 and MPO-ANCA not only have a well-defined genetic basis but also because it confers a disease specificity that IIF testing lacks. Not all patients who have pathological evidence of ANCA vasculitis are ANCA-positive. About 9-17% of patients with AAV test negative for ANCA and more than 50% of patients with Churg-Strauss syndrome are ANCA-negative [16].

The treatment of AAV typically involves an induction phase, maintenance of remission phase, and treatment of relapses. The standard for induction therapy for AAV has traditionally been a combination of corticosteroids with cyclophosphamide for a period of 3-6 months. However, the toxicity of cumulative doses of cyclophosphamide has always been a source of concern. The Pulse Versus Daily Oral Cyclophosphamide for Induction of Remission in Antineutrophil Cytoplasmic Antibody-Associated Vasculitis Study (CYCLOPS) demonstrated that monthly intravenous pulse doses of cyclophosphamide achieved similar remission rates when compared to daily oral cyclophosphamide while reducing cumulative total dose [18]. Rituximab could also be used as an alternative to cyclophosphamide in the remission induction for AAV.

In non-organ threatening AAV, methotrexate and mycophenolate mofetil (MMF) have been shown to be equally effective in inducing remission. For maintenance of remission, azathioprine is the medication of choice in combination with low-dose steroids according to EULAR/ERA-EDTA [19]. Other medications that can be used in remission maintenance include MMF, methotrexate, and rituximab. Relapses in AAV are common and rituximab has been shown in the RITUXVAS trial to be superior to cyclophosphamide in the treatment of relapses [20].

We hypothesize that the occurrence of new-onset ANCA-vasculitis in our patient is associated with tofacitinib because there have been published case reports of increased risk of inducing vasculitis in patients treated with tyrosine-kinase inhibitors [21-23]. Our patient had tested negative for P-ANCA prior to treatment with tofacitinib and subsequently became p-ANCA positive with biopsy-confirmed ANCA-vasculitis disease after treatment with tofacitinib. In the opinion of the authors, the mechanism of action of tofacitinib which involves activation by myeloperoxidase in neutrophils may have an association with the induction of ANCA-vasculitis. With the increasing body of evidence demonstrating an association between tyrosine-kinase inhibitors and induction of vasculitis, a randomized-control trial is warranted to confirm a causal relationship.

## Conclusions

Tofacitinib has increasingly been used in the United States since it was first approved for use in 2012. The common side effects of neutropenia, infections are well known and expected. This case of vasculitis may have been induced by tofacitinib, but a clear causality can't be proven based on this case report. It is unique because tofacitinib is used to treat an over-active immune system but in our patient, it also induced another disease of the immune system. It also raises the question of whether the mechanism of activation of tofacitinib by myeloperoxidase in neutrophils has some relationship with the occurrence of new-onset ANCA-vasculitis. Clinicians should therefore have a high index of suspicion with the onset or worsening of proteinuria and/or hematuria in a patient being treated with tofacitinib.
